# The triglyceride-glucose index predicts peripheral artery disease complexity

**DOI:** 10.3906/sag-2006-180

**Published:** 2020-08-26

**Authors:** Bilge DURAN KARADUMAN, Hüseyin AYHAN, Telat KELEŞ, Engin BOZKURT

**Affiliations:** 1 Department of Cardiology, Faculty of Medicine, Atılım University, Medicana International Ankara Hospital, Ankara Turkey; 2 Department of Cardiology, Faculty of Medicine, Ankara Yıldırım Beyazıt University, Ankara City Hospital, Ankara Turkey; 3 Department of Cardiology, Medicana International Ankara Hospital, Ankara Turkey

**Keywords:** Peripheral arterial disease, triglyceride, glucose, atherogenic index, TASC-II classification

## Abstract

**Background/aim:**

High levels of triglyceride (TG) and fasting blood glucose (FBG) values increase atherosclerosis risk. This study evaluates the relationship between peripheral artery disease (PAD) severity and complexity, as assessed by TransAtlantic InterSociety Consensus-II (TASC-II) classification and the triglyceride-glucose (TyG) index.

**Materials and methods:**

A total of 71 consecutive patients with PAD (males 93%, mean age 63.3 ± 9.7), who underwent percutaneous peripheral intervention were included retrospectively. The patients were divided into two groups according to the angiographically detected lesions. Those with TASC A-B lesions were included in Group 1, and those with TASC C-D lesions were included in Group 2. TyG index was calculated as formula: ln[fasting TG (mg/dL) × fasting plasma glucose (mg/dL)/2].

**Results:**

There were 40 patients in Group 1 (90.3% men, with a mean age of 63.6 ± 9.3 years) and 31 patients in Group 2 (96.8% men, with a mean age of 62.0 ± 8.6 years). In the majority of patients in both groups, the target vessels are iliac arteries and femoral arteries. In Group 2, platelet count and TyG index were significantly high, according to Group 1. The TyG index was significantly correlated with TASC-II, Rutherford category, HbA1c, and HDL-C.

**Conclusion:**

In this present study, we showed that the TyG index was an independent predictor of peripheral artery disease complexity, according to TASC-II classification, for the first time in the literature.

## 1. Introduction

Peripheral arterial disease (PAD) is an essential cause of cardiovascular morbidity and mortality worldwide, and its frequency is gradually increasing. Although lower extremity peripheral artery disease is more common in men, its incidence also increases in women over 50 years. The presentations of PAD can differ from asymptomatic to intermittent claudication, critical limb ischemia (CLI), or acute limb ischemia (ALI) [1]. Since the underlying pathology is atherosclerosis, its association with ischemic heart diseases and cerebrovascular diseases is common [2]. Risk factors for atherosclerosis, for instance, hypertension (HT), diabetes mellitus (DM), smoking, hyperlipidemia (HL), and obesity, are common among patients with PAD [3]. PAD is associated with an increased risk of cardiovascular and all-cause mortality as well [4]. 

It is remarkably relevant to define the complexity of the PAD to make an early diagnosis and design the treatment strategy in patients with clinical suspicion. TransAtlantic InterSociety Consensus-II (TASC-II) classification is a consensus definition that is used to evaluate PAD concerning the anatomic distribution of lesions. This anatomical classification provides the most appropriate revascularization strategy (endovascular and surgical) and treatment decisions based on the complexity, number, and location of the lesions [5].

High levels of triglyceride (TG) and fasting blood glucose (FBG) are the most critical risk factors for cardiovascular disease (CVD) [6]. The combination of both indicators, triglyceride glucose (TyG) index, is a novel marker, which has been verified to have a high sensitivity and specificity in identifying metabolic syndrome [7]. Furthermore, studies have shown an association of the TyG index with the incidence of CVD [8], stroke [9], carotid atherosclerosis [10], and CAD incidence [11]. Unfortunately, there is currently insufficient data on whether the TyG index predicts the severity and complexity of peripheral artery disease. Therefore, in our study, we aimed to evaluate the relationship between PAD severity and complexity evaluated by TASC-II classification and TyG ratio.

## 2. Materials and methods

A total of 71 consecutive patients with PAD (males 93%, mean age 63.3 ± 9.7), who underwent percutaneous peripheral intervention in our tertiary care center were included retrospectively. All demographic data (age, sex), comorbidities (DM, HT, hyperlipidemia, smoking, atrial fibrillation, CAD), physical conditions, symptoms, and routine blood tests were obtained from hospital records. Classification of PAD was performed due to TASC II guidelines. All patients were divided into two groups due to the severity of the lesions detected angiographically. Those with TASC A-B lesions were included in Group 1, and those with TASC C-D lesions were included in Group 2. According to the current literature, Rutherford and Fontaine’s classification was obtained for each patient [12].

Peripheral blood samples were obtained from the antecubital vein after at least 12-h fasting for detection of complete blood count (CBC), total serum cholesterol (TC), triglycerides (TGs), HDL-cholesterol, and low-density lipoprotein (LDL) cholesterol and plasma glucose (Sysmex K-1000, Sysmex Corporation, Kobe, Japan) on admission. All routine biochemical analyses were performed using an auto-analyzer (Roche Diagnostic Modular Systems, Tokyo, Japan). TyG index was calculated as formula: ln[fasting TG (mg/dL) × fasting plasma glucose (mg/dL)/2]. The local ethics committee approved this study, and individual informed consent was obtained from all patients.

### 2.1. Statistical analysis 

Data are expressed as percentages or mean ± standard deviation. Continuous variables were checked for normal distribution assumptions using Kolmogorov–Smirnov test. Continuous variables were presented as the mean ± standard deviation and compared using t-tests (for data complying with a normal distribution) or Mann–Whitney U-tests (for data complying with nonnormal distribution). Categorical variables were presented as frequencies and percentages and compared using Pearson’s and Fisher’s exact test the chi-square test. Correlation analysis was performed using Pearson or Spearman tests. All analyses were performed using SPSS for Windows version 22.0 (IBM Corp., Armonk, NY, USA). A P-value <0.05 was considered as signiﬁcant.

## 3. Results

The demographic, laboratory and procedural characteristics of patients were categorized by TASC-II classification. There were 40 patients in Group 1 (90.3% men, with a mean age of 63.6 ± 9.3 years) and 31 patients in Group 2 (96.8% men, with a mean age of 62.0 ± 8.6 years). Table 1 shows the comparison of clinical characteristics between the two groups. While most of the patients in both groups presented with intermittent claudication symptoms, 9.9% of all patients consulted with rest pain. As expected, walking distance without claudication was increased in group 1 (99.4 ± 67.8 vs. 61.7 ± 53.6, P: 0.018). 32.5% of patients in group 1 previously had a history of CABG and was statistically significantly more common in patients than group 2 (P: 0.008). According to the Fontaine and Rutherford classification, patients in Group 2 were more severely symptomatic (P: 0.004 vs. P: 0.006, respectively).

**Table 1:** Baseline characteristics of the patients.

Parameters	Group 1 n = 40	Group 2 n = 31	P value
Age (years)	63.6 ± 9.3	62.0 ± 8.6	0.465
Male n (%)	36 (95.0)	28 (90.3)	0.445
Symptom n (%) - Intermittent Claudication - Rest pain - Trophic Changes	35 (87.5) 1 (2.5) 4 (10.0)	24 (77.4) 4 (12.9) 3 (9.7)	0.287
Walking distance without claudication (mt)	99.4 ± 67.8	61.7 ± 53.6	0.018
HT n (%)	35 (87.5)	25 (80.6)	0.429
HL n (%)	20 (50.0)	15 (48.4)	0.324
Current smoker n (%)	19 (47.5)	15 (48.4)	0.288
DM n (%)	17 (52.5)	21 (67.7)	0.079
AF n (%)	1 (2.5)	3 (9.7)	0.193
Previous PCI n (%)	18 (45.0)	10 (32.3)	0.276
Previous CABG n (%)	13 (32.5)	2 (6.5)	0.008
Previous peripheral intervention n (%)	9 (22.5)	2 (6.5)	0.061
Stenosis (%)	92.6 ± 7.5	95.7 ± 6.7	0.094
CAD n (%) - Normal - Nonobstructive - 1 vessel disease - 2 vessel disease - 3 vessel disease	1 (2.5) 6 (15.0) 12 (30.0) 18 (45.0) 3 (7.5)	1 (3.2) 5 (16.1) 10 (32.3) 12 (38.7) 3 (9.7)	0.987
Rutherford category n (%) - 2 - 3 - 4 - 5	14 (38.9) 17 (47.2) 4 (11.1) 1 (2.8)	2 (6.9) 14 (48.3) 6 (20.7) 7 (27.1)	0.004
Fontaine grade n (%) - I - IIA - IIB - III - IV	1 (2.8) 6 (16.7) 15 (41.7) 13 (36.1) 1 (2.8)	0 1 (3.4) 5 (17.2) 15 (51.7) 8 (27.6)	0.006
Target vessel n (%) - CIA - SFA - Popliteal - Below the knee	16 (40.0) 17 (42.5) 5 (12.5) 2 (5)	14 (45.1) 13 (41.9) 2 (6.4) 2 (6.4)	0.442
Peripheral intervention n (%) - PTA - Stent - PTA+Stent - Drug eluting balloon	11 (28.2) 7 (17.9) 21 (53.8) 11 (28.2)	6 (22.2) 2 (7.4) 19 (70.4) 5 (18.5)	0.326
Type of stent n (%) - Self -expandable - Balloon expandable	15 (55.6) 12 (44.4)	11 (52.4) 10 (47.6)	0.827
Mean balloon diameter mm	5.2 ± 1.3	4.9 ± 1.7	0.503
Mean balloon length mm	62.6 ± 43.5	63.8 ± 37.0	0.917
Mean stent diameter mm	7.4 ± 1.1	7.2 ± 1.3	0.509
Mean stent length mm	56.1 ± 36.8	59.2 ± 33.8	0.714
LVEF (%)	51.3 ± 13.8	50.3 ± 14.6	0.776
LVEDD (cm)	5.0 ± 0.8	4.8 ± 0.4	0.286
LVESD (cm)	3.3 ± 1.0	3.2 ± 0.6	0.870
LA (cm)	4.1 ± 0.4	3.7 ± 0.4	<0.001
sPAP (mmHg)	22.5 ± 12.8	25.3 ± 16.8	0.467

HT: Hypertension; HL: Hyperlipidemia; DM: Diabetes mellitus; AF: Atrial fibrillation; PCI: Percutaneous coronary intervention, CABG: Coronary artery bypass grafting, CAD: Coronary artery disease, CIA: Common iliac artery, SFA: Superficial femoral artery; PTA: Percutaneous transluminal angioplasty; LVEF: Left ventricular ejection fraction, LVEDD: Left ventricular end diastolic diameter, LVESD: Left ventricular end systolic diameter, LA: Left atrium, sPAP: Systolic pulmonary artery pressure.

In the majority of patients in both groups, the target vessels were iliac arteries and femoral arteries. The vast majority of patients in both groups were treated with percutaneous transluminal angioplasty and stent implantation. There was no difference between the two groups in terms of stent type, mean balloon diameter, mean balloon length, mean stent diameter, and mean stent length. 

The laboratory variables of all patients are presented in Table 2. The groups were similar in terms of hemoglobin, serum glucose, HbA1c, creatinine, C-reactive protein, total cholesterol, triglyceride, high-density lipoprotein cholesterol (HDL-C), and low-density lipoprotein cholesterol (LDL-C) values. In Group 2, platelet count and TyG index were significantly high, according to Group 1 (P: 0.035, P: 0.011, respectively).

**Table 2:** Laboratory variables of the patients.

Parameters	All Patients n = 71	Group 1 n = 40	Group 2 n = 31	P value
Serum glucose (mg/dL)	127.8 ± 69.0	113.5 ± 55.5	142.9 ± 80.6	0.077
HbA1c (%)	6.55 ± 1.46	6.39 ± 1.01	6.89 ± 1.98	0.480
Serum creatinine (mg/dL)	1.1 ± 0.8	1.0 ± 0.6	1.2 ± 0.9	0.288
Total cholesterol (mg/dL)	191.3 ± 43.7	191.1 ± 46.6	184.8 ± 39.1	0.563
Triglyceride (mg/dL)	182.4 ± 106.5	160.3 ± 62.9	212.2 ± 147.8	0.052
LDL cholesterol (mg/dL)	116.0 ± 36.3	111.1 ± 37.7	116.2 ± 32.8	0.565
HDL cholesterol (mg/dL)	39.7 ± 12.1	38.7 ± 11.0	40.3 ± 14.4	0.599
Hemoglobin g/dL	14.5 ± 2.0	14.5 ± 2.1	14.8 ± 1.9	0.428
Platelet ×10^9^ /L	239.5 ± 59.7	225.2 ± 58.3	255.9 ± 60.2	0.035
TyG	73575.0 ± 13069.1	9019.8 ± 6096.8	18209.4 ± 20873.9	0.011

HbA1c: Glycated hemoglobin, LDL: Low-density lipoprotein, HDL: High-density lipoprotein, TyG: Triglyceride-glucose index.

Correlation analysis was used to examine the relationship between the TyG index and clinical variables. The TyG index was significantly correlated with TASC-II, Rutherford category, HbA1c, and HDL-C (Table 3). 

**Table 3:** Correlation between the TyG index and clinical variables.

Parameters	r value	P value
Stenosis	0.090	0.445
Age	-0.079	0.495
Walking distance without claudication	-0.168	0.178
TASC-II	0.302	0.011
Rutherford category	0.249	0.047
Fontaine grade	0.199	0.115
HbA1c	0.647	<0.001
HDL-C	-0.315	0.007

HbA1c: Glycated hemoglobin, HDL: High-density lipoprotein, TyG: Triglyceride-glucose index.

## 4. Discussion

This is the first study to analyze the association between the TyG index and PAD severity to the best of our knowledge. The main findings are as follows: (1) the TyG index is an independent predictor of peripheral artery disease complexity and (2) the TyG index is correlated with TASC-II classification, Rutherford category, and also HDL-C.

The TyG index is a composite indicator consisting of TG and fasting blood glucose (FBG), and is a useful marker of insulin resistance (IR) and a predictor of type 2 diabetes mellitus (T2DM) [13]. Afterward, several studies were managed and found a positive relationship between the TyG index and CVD. Irace et al. evaluated the relationship between carotid atherosclerosis and the TyG index in two different groups and gained positive results [10]. Besides, a study involving 4319 patients showed that the TyG index was significantly associated with the presence of coronary calcification [14]. Several prospective studies have been conducted on the link between the TyG index and cardiovascular events (CVEs). Vega et al. investigated the relationship between TyG index and cardiovascular mortality, CAD or CVD in 39,447 men and showed that the TyG index does not predict CVD mortality, but this study does not reflect the general population [15]. In another study, higher levels of the TyG index in 5014 healthy individuals were associated with an increased risk of developing CVD [16]. In another observational study, Mao et al. showed that the TyG index might be an independent predictor of coronary artery disease severity and cardiovascular outcomes in acute coronary syndrome [16]. But there is no study about the relationship between TyG index and PAD severity. Currently, only a few studies have been reported on the association between the TyG index and CVD; however, there is no study investigating its relationship with peripheral artery disease complexity.

The role of blood lipid parameters in the development of atherosclerotic PAD is crucial. Epidemiological studies have shown that dyslipidemia alone is sufficient for atherosclerosis development, even without other risk factors [17]. In current studies, Kim et al. [14], and Lee et al. [18] reported that the TyG index was linked with arterial stiffness and coronary artery calcification in Korean adults. Another abovementioned study [16] found that the TyG index was a predictor of hypertension in a Chinese population. All these studies implied that the TyG index could act as a biomarker for arterial diseases, and the IR predicted by the TyG index may have been involved in vascular remodeling and atherogenesis. 

But despite all these studies, the relationship between the TyG index and peripheral artery disease complexity remains unclear. The TyG index formula consists of a combination of TG and fasting blood glucose. TG’s relationship with the risk of CVD is still controversial, but a new set of evidence has proved that TG and TG-rich lipoproteins are the causative factors of CVD [19]. The concurrency of hypertriglyceridemia (HTG) promotes the formation of small dense LDL particles [20]. Although most studies have evaluated TG’s risk of CVD in HTG patients only, several studies have shown that high-normal range plasma TG predicts CVEs. Glucose disorder is another CVD risk factor that often coexists with HTG. Evaluating these two parameters together increases their CVE predictive power. Since atherosclerosis is a common etiopathogenesis for CVD and PAD, the TyG index’s analysis was decided in our study.

The TASC-II guidelines were published in 2007 and involved a change of the original TASC classification for PAD, focusing on the aortoiliac and femoropopliteal regions. TASC-II also aimed to guide treatment decisions correlating to the optimal revascularization strategy (endovascular vs. surgical) according to the patient’s anatomic and clinical status. Generally, this revision issued in the reclassification of more complex anatomies into less severe categories of the TASC classification (e.g., TASC C lesions reclassified as TASC B lesions with an associated alteration from surgical to endovascular treatment). TASC A and B lesions were still suggested for primary endovascular revascularization, TASC D lesions for surgical revascularization, and TASC C lesions for surgical revascularization in patients with proper perioperative risk and accessible conduit [21]. According to recent studies, the increase in TG/HDL ratio, also known as the atherogenic index, is a significant risk factor for cardiovascular diseases and metabolic syndrome [22]. Mesut et al. [23] showed that the TG/HDL-C ratio could predict the angiographic complexity of peripheral artery disease according to the TASC II classification. Based on these data, it is not surprising to predict the peripheral artery disease complexity with the TyG index, as we showed in our study.

There are several limitations to our study. First of all, this study is a single-center retrospective trial, and the sample size is small. Another limitation of the study is the absence of a control group. Since our center is a reference center, as long-term follow-up data is limited, the effect on mortality is uncertain. Further multicenter prospective studies may generate more relevant results on this issue.

In this present study, we showed that the TyG index was an independent predictor of peripheral artery disease complexity, according to TASC-II classification, for the first time in the literature. TyG index is a simple parameter that can be easily obtained from routine biochemical parameters, and large-scale studies are needed to evaluate whether it predicts PAD complexity.
